# Legionella-Associated Encephalopathy With a Reversible Splenial Lesion and Additional Reversible Cortical and White Matter Lesions

**DOI:** 10.7759/cureus.116612

**Published:** 2026-09-20

**Authors:** Yuki Higashi, Taku Mayahara, Tomohiro Katayama

**Affiliations:** 1 Department of Emergency and General Medicine, Kobe Ekisaikai Hospital, Kobe, JPN

**Keywords:** cytotoxic lesion of the corpus callosum, diffusion-weighted imaging, hyponatremia, legionella pneumophila, mild encephalitis/encephalopathy with a reversible splenial lesion

## Abstract

Cytotoxic lesions of the corpus callosum (CLOCCs) are diffusion-restricted callosal lesions associated with various infectious and metabolic conditions. The clinicoradiological syndrome traditionally termed mild encephalitis/encephalopathy with a reversible splenial lesion (MERS) substantially overlaps with this imaging concept. We describe the case of a 60-year-old man with *Legionella* pneumonia and marked hyponatremia (serum sodium, 121 mEq/L) whose neurological symptoms progressed to incoherent speech and gait disturbance. Brain magnetic resonance imaging (MRI) on hospital day 3 demonstrated a diffusion-restricted lesion in the splenium of the corpus callosum together with additional unilateral lesions involving the right temporo-occipital cortex and white matter. All three lesions showed restricted diffusion with reduced apparent diffusion coefficient values. With treatment of *Legionella* pneumonia and correction of hyponatremia, the neurological abnormalities resolved, and follow-up MRI demonstrated complete resolution of all lesions. The splenial lesion was consistent with a CLOCC, whereas the unilateral cortical and white matter lesions were atypical of the conventional pattern described as MERS type 2. This case illustrates that reversible callosal, cortical, and white matter MRI abnormalities may occur in the setting of *Legionella* pneumonia and marked hyponatremia, although a common pathophysiology cannot be established.

## Introduction

Cytotoxic lesions of the corpus callosum (CLOCCs) are diffusion-restricted lesions of the corpus callosum associated with a wide variety of infectious, metabolic, toxic, and other systemic conditions [1]. Magnetic resonance imaging (MRI) typically demonstrates splenial involvement, with hyperintensity on diffusion-weighted imaging (DWI) and reduced apparent diffusion coefficient (ADC) values. The clinicoradiological syndrome traditionally termed mild encephalitis/encephalopathy with a reversible splenial lesion (MERS) overlaps substantially with the CLOCC concept and is characterized by transient neurological symptoms and a reversible splenial lesion [2,3]. Hyponatremia has been proposed as an important contributing factor in the development of these lesions [4].

Neurological manifestations are well recognized in Legionnaires’ disease. In an early clinical series and literature review, neurological abnormalities were reported in approximately 40-50% of patients, most commonly as altered mental status, with cerebellar dysfunction and focal neurological deficits reported less frequently [5]. *Legionella pneumophila* infection has also occasionally been associated with reversible splenial lesions [6]. In the traditional MERS classification, type 2 refers to cases in which lesions extend beyond the splenium, predominantly involving other portions of the corpus callosum or bilateral symmetric cerebral white matter [7]. Cortical gray matter involvement is therefore not typical of the classical type 2 pattern. Nevertheless, a previous report of *Legionella*-associated CLOCC described restricted diffusion in the bilateral cerebral cortex in addition to a splenial lesion [8], indicating that cortical diffusion abnormalities may also occur in this clinical setting.

Here, we report the case of a patient with *Legionella *pneumonia and marked hyponatremia in whom a splenial CLOCC and separate unilateral cortical and white matter lesions with restricted diffusion were detected during the same acute illness. Because both acute infection and marked hyponatremia were present and alternative neurological mechanisms could not be completely excluded, the causal relationship between these factors and the MRI abnormalities remains uncertain. This case highlights an unusual combination of reversible callosal, cortical, and white matter abnormalities occurring in this clinical setting.

## Case presentation

A 60-year-old man with a history of diabetes mellitus and hypertension had experienced several days of malaise, cough, and fever and was on his way to the hospital for evaluation. While getting off a bus, he became unsteady and fell, striking the right frontal region of his head. Emergency medical personnel documented no loss of consciousness or vomiting. He sustained a minor right frontal scalp laceration that did not require suturing.

On arrival, he was febrile and mildly disoriented; his level of consciousness was Japan Coma Scale (JCS) I-2 [9], with a Glasgow Coma Scale (GCS) score of 14 (E4V4M6). His pupils were equal and reactive to light. No limb weakness, sensory deficit, ocular motility disturbance, dysarthria, or aphasia was identified, and he was able to follow simple commands. His temperature was 38.7°C, blood pressure was 129/71 mmHg, heart rate was 100 beats/minute, and oxygen saturation was 97% on room air. Laboratory testing showed marked hyponatremia (serum sodium, 121 mEq/L), an elevated C-reactive protein level (25.4 mg/dL), and a white blood cell count of 8,300/μL (Table 1). Thrombocytopenia (platelet count, 8.0 × 10⁴/μL) was noted on admission together with an elevated D-dimer level (5.2 μg/mL). Infection-associated coagulopathy was considered, but no clinically significant bleeding or thrombotic event occurred. The thrombocytopenia was transient and resolved as the systemic condition improved.

**Table 1:** Laboratory findings on admission and cerebrospinal fluid examination on hospital day 3 Blood and urine test results were obtained on admission (hospital day 1). CSF results were obtained on hospital day 3 at the time of lumbar puncture. eGFR, estimated glomerular filtration rate; AST, aspartate aminotransferase; ALT, alanine aminotransferase; ALP, alkaline phosphatase; LDH, lactate dehydrogenase; γ-GTP, gamma-glutamyl transpeptidase; HbA1c, hemoglobin A1c; PT, prothrombin time; INR, international normalized ratio; APTT, activated partial thromboplastin time; CSF, cerebrospinal fluid.

Parameter	Result	Reference range	Unit
White blood cell count	8.3	3.5–9.0	×10³/μL
Red blood cell count	471	380–530	×10⁴/μL
Hemoglobin	14.7	12–17	g/dL
Hematocrit	41.4	32–49	%
Platelet count	8.0	15–35	×10⁴/μL
Sodium	121	135–147	mEq/L
Potassium	3.3	3.6–5.0	mEq/L
Chloride	90	98–108	mEq/L
Urine sodium	<20	—	mEq/L
Urine chloride	25	—	mEq/L
Blood urea nitrogen	21	8–20	mg/dL
Creatinine	1.34	0.8–1.2	mg/dL
eGFR	43.5	≥60	mL/min/1.73 m²
Total bilirubin	1.7	0.2–1.2	mg/dL
Total protein	6.2	6.5–8.3	g/dL
Albumin	3.3	3.8–5.3	g/dL
AST	67	≤35	U/L
ALT	53	≤35	U/L
ALP	53	38–113	U/L
LDH	311	121–230	U/L
γ-GTP	18	≤50	U/L
Creatine kinase	367	30–200	U/L
Calcium	7.9	8.5–10.2	mg/dL
C-reactive protein	25.4	≤0.5	mg/dL
Glucose	118	70–110	mg/dL
HbA1c (NGSP)	7.2	4.6–6.2	%
PT	72	70–140	%
PT-INR	1.18	—	—
APTT	36.4	≤35	s
D-dimer	5.2	<1.0	μg/mL
CSF cell count	10	0–5	/μL
CSF protein	56	10–40	mg/dL
CSF glucose	37	40–75	mg/dL
CSF chloride	109	120–125	mmol/L

Head computed tomography (CT) showed no intracranial hemorrhage or skull fracture. Chest CT revealed pneumonia predominantly involving the left lower lobe. Urinary antigen testing was positive for *L. pneumophila*, and *Legionella* pneumonia was diagnosed. Intravenous azithromycin was initiated on hospital day 1. Hyponatremia was treated with 1,500 mL/day of a maintenance crystalloid solution containing 35 mEq/L of sodium, supplemented with 40 mL/day of 10% sodium chloride. The serum sodium concentration increased from 121 mEq/L on admission to 127 mEq/L on hospital day 2, was not measured on hospital day 3, remained 127 mEq/L on hospital day 4, and normalized to 135 mEq/L by hospital day 15.

The altered mental status on presentation was initially considered mild and potentially attributable to fever and hyponatremia. However, his neurological symptoms became more prominent after admission. By hospital day 3, his level of consciousness remained at JCS I-2, but he had developed incoherent speech and an unsteady gait and responded sluggishly to commands. Limb strength remained preserved (Medical Research Council (MRC) Scale for Muscle Strength [10] grade 5/5 in all extremities), and no clear focal motor deficit was documented. Brain MRI performed on hospital day 3 demonstrated a hyperintense lesion in the splenium of the corpus callosum on DWI. The splenial lesion showed subtle corresponding hypointensity on the ADC map and faint hyperintensity on fluid-attenuated inversion recovery (FLAIR) imaging, consistent with a CLOCC (Figure 1A-1C).

**Figure 1:**
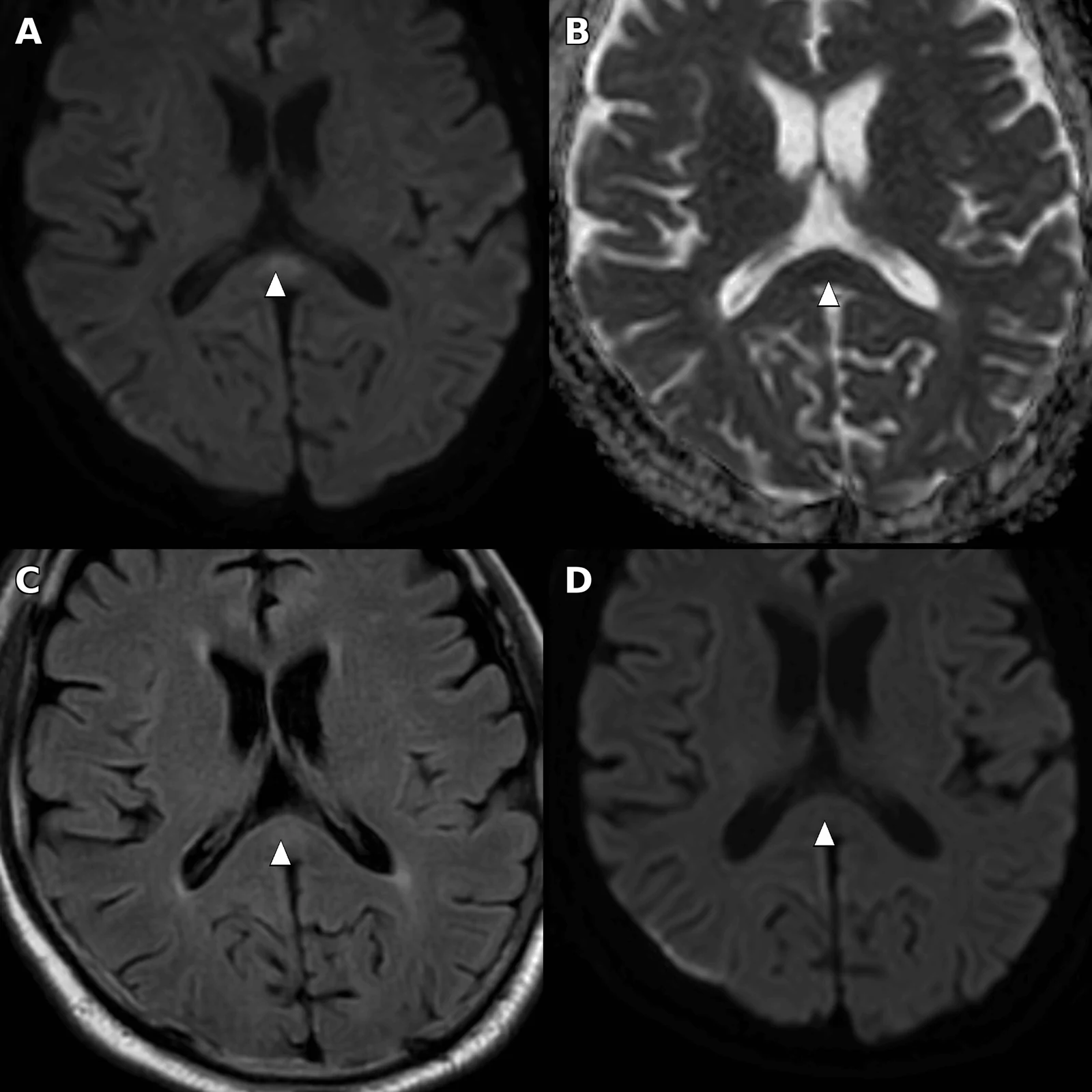
Magnetic resonance imaging findings of the splenial lesion. On hospital day 3, diffusion-weighted imaging (DWI) showed a hyperintense lesion in the splenium of the corpus callosum (A), with subtle corresponding hypointensity on the apparent diffusion coefficient (ADC) map (B) and faint hyperintensity on fluid-attenuated inversion recovery (FLAIR) imaging (C). Follow-up DWI on hospital day 13 showed complete resolution of the splenial lesion (D). Arrowheads indicate the lesion in (A-C) and its prior location in (D).

In addition, DWI revealed two hyperintense lesions in the right temporo-occipital region. One involved the cortical ribbon on superior slices, whereas the other involved the white matter on more inferior slices. Both lesions were remote from the right frontal impact site, and no corresponding abnormality was identified on the initial head CT or T2*-weighted imaging. Both extrasplenial lesions showed corresponding low signal on ADC maps. The cortical lesion showed faint corresponding hyperintensity on FLAIR imaging, whereas no definite corresponding FLAIR abnormality was identified in the white matter lesion (Figure 2).

**Figure 2:**
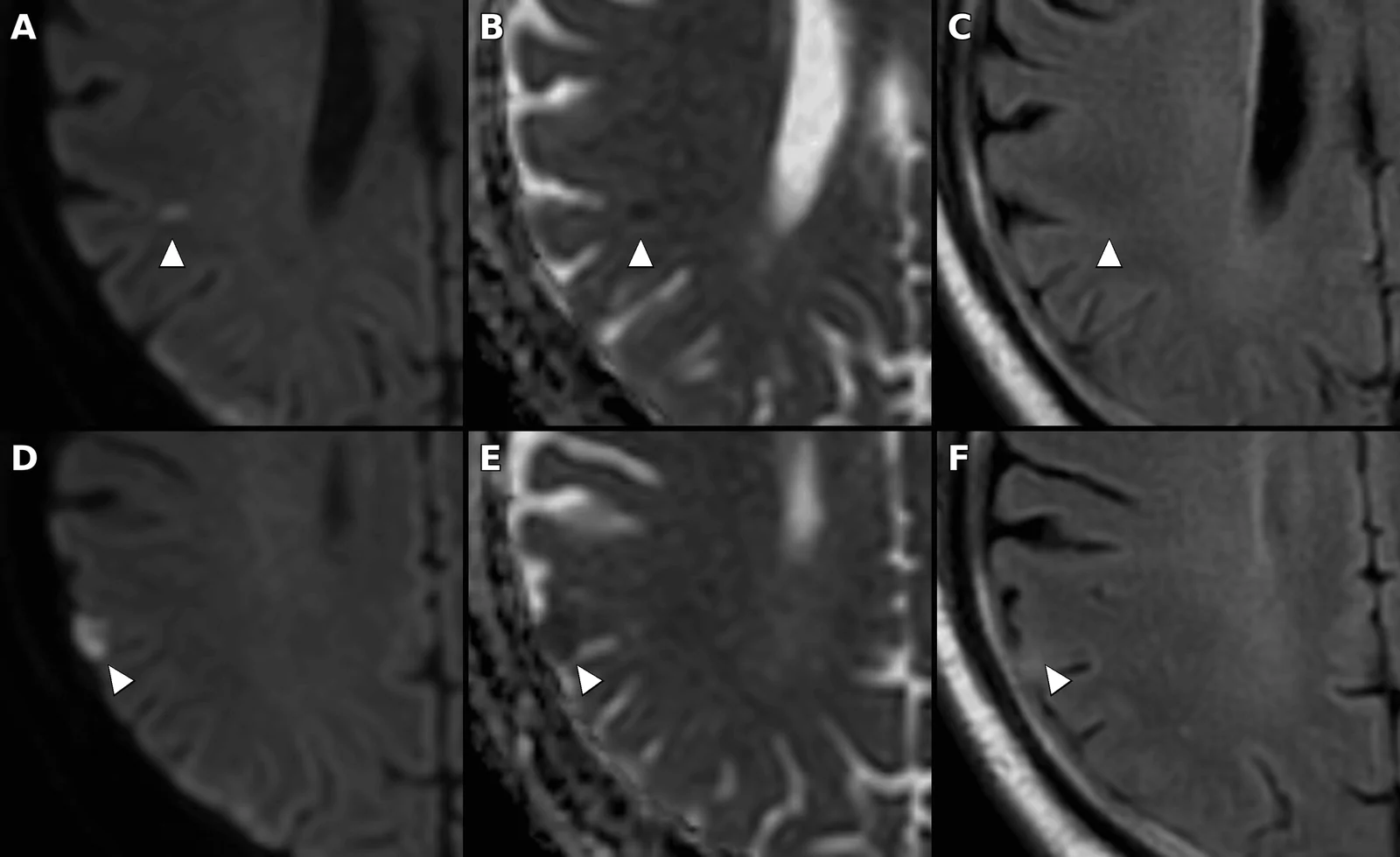
Magnetic resonance imaging findings of the extrasplenial lesions on hospital day 3. A right temporo-occipital white matter lesion was hyperintense on diffusion-weighted imaging (DWI) (A) with corresponding hypointensity on the apparent diffusion coefficient (ADC) map (B) and no definite corresponding abnormality on fluid-attenuated inversion recovery (FLAIR) imaging (C). A separate right temporo-occipital cortical lesion was also hyperintense on DWI (D) with corresponding hypointensity on the ADC map (E) and faint corresponding hyperintensity on FLAIR imaging (F). Arrowheads indicate the lesions in (A), (B), (D), (E), and (F) and the corresponding location in (C).

Quantitative region-of-interest analysis of the ADC map demonstrated lower ADC values in all three lesions than in their respective non-lesional reference regions. The ADC value was 0.57 × 10⁻³ mm²/s in the splenial lesion compared with 0.84 × 10⁻³ mm²/s in the anterior non-lesional corpus callosum, 0.61 × 10⁻³ mm²/s in the cortical lesion compared with 0.86 × 10⁻³ mm²/s in the contralateral homologous cortex, and 0.45 × 10⁻³ mm²/s in the white matter lesion compared with 0.80 × 10⁻³ mm²/s in the contralateral homologous white matter. 

Lumbar puncture was performed on hospital day 3. Cerebrospinal fluid (CSF) examination showed mild pleocytosis, with a cell count of 10 cells/μL (50% polymorphonuclear leukocytes and 50% mononuclear cells), a protein concentration of 56 mg/dL, and a glucose concentration of 37 mg/dL (serum glucose, 76 mg/dL; CSF-to-serum glucose ratio, 0.49). Opening pressure was not measured. Gram stain and routine CSF culture were negative. CSF testing for *Legionella* by polymerase chain reaction (PCR) or culture was not performed, nor was viral PCR testing.

With continued treatment of *Legionella* pneumonia and correction of hyponatremia, the patient's neurological symptoms gradually improved. Follow-up MRI on hospital day 13 showed resolution of the splenial and white matter lesions, whereas the cortical lesion remained visible on DWI (Figure 1D, 3A, 3B). By hospital day 15, his mental status and gait had returned to normal. He was discharged on hospital day 24 without apparent neurological sequelae. Outpatient follow-up MRI on day 33 after admission demonstrated complete resolution of the remaining cortical DWI abnormality (Figure 3C), with no definite residual abnormality on the corresponding FLAIR image (Figure 3D).

**Figure 3:**
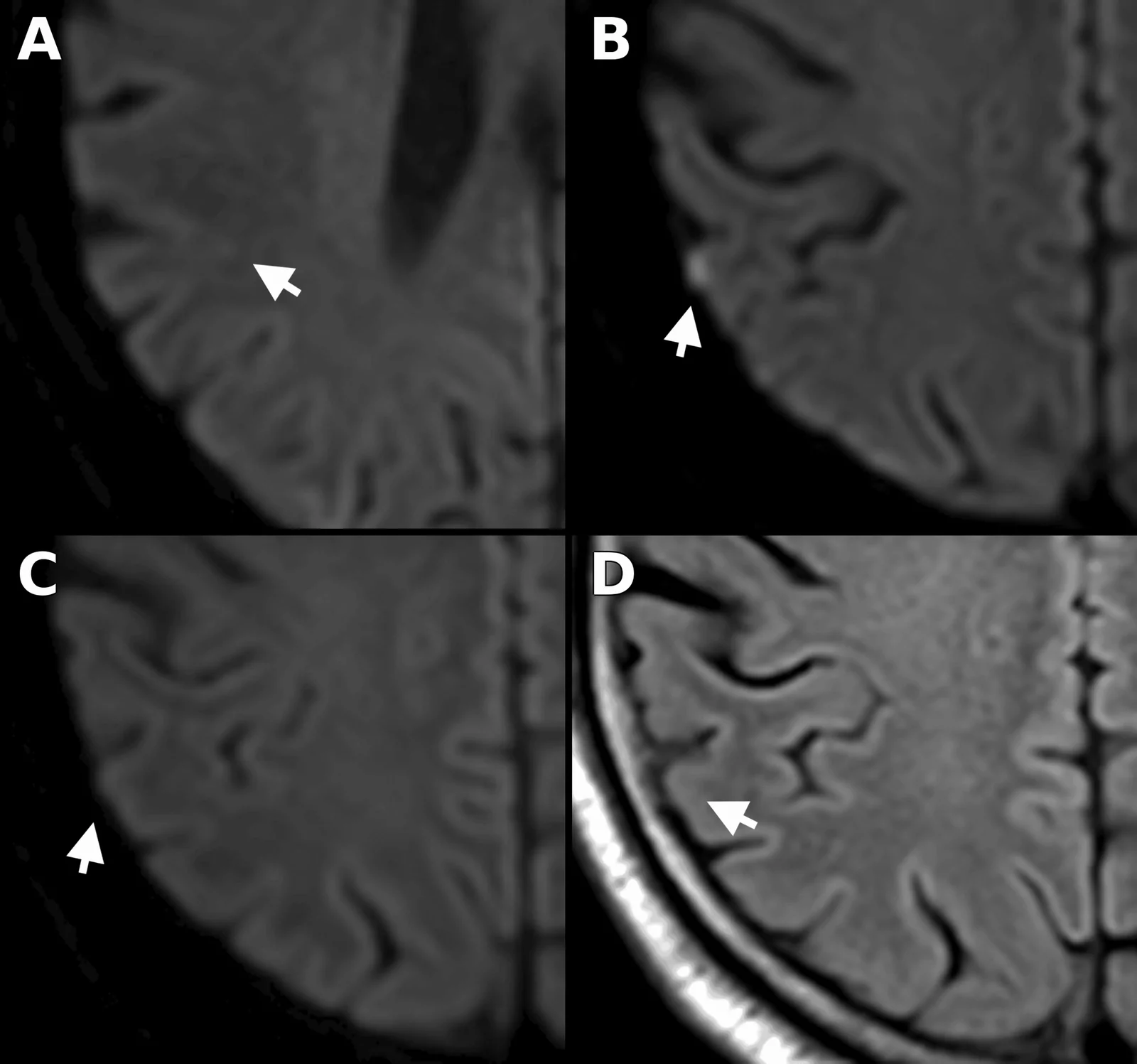
Serial magnetic resonance imaging findings of the extrasplenial lesions. On hospital day 13, the previously observed right temporo-occipital white matter lesion had resolved on diffusion-weighted imaging (DWI) (A), whereas the cortical lesion remained visible on DWI (B). On day 33, the cortical DWI abnormality had completely resolved (C), with no definite residual abnormality on the corresponding fluid-attenuated inversion recovery (FLAIR) image (D). Arrowheads indicate the prior location of the white matter lesion in (A), the residual cortical lesion in (B), and the corresponding location of the previously observed cortical lesion in (C) and (D).

## Discussion

This case demonstrated a splenial CLOCC together with unilateral cortical and white matter lesions in a patient with *Legionella* pneumonia and marked hyponatremia. All three lesions showed restricted diffusion with corresponding reductions in ADC values and ultimately resolved. The cortical and white matter abnormalities were unusual because their distribution differed from the predominantly callosal and bilateral symmetric white matter pattern traditionally described as MERS type 2.

The relationship between CLOCCs and MERS is primarily one of overlapping radiological and clinical concepts [1,3]. CLOCC describes the callosal lesion itself without assuming a particular clinical severity or outcome, whereas MERS has traditionally been used for a clinicoradiological syndrome involving a reversible splenial lesion [2,3]. In classical MERS type 2, additional abnormalities predominantly involve other portions of the corpus callosum or bilateral symmetric cerebral white matter [7]. The unilateral cortical and white matter lesions in our patient therefore cannot simply be regarded as a typical type 2 distribution.

Published cases and reviews of *Legionella*-associated MERS/CLOCC have predominantly described an isolated reversible splenial or callosal lesion [6,11,12]. Raybould et al. reviewed earlier cases of Legionnaires’ disease associated with reversible splenial lesions [11]. More recently, Kageyama et al. reviewed adult-onset MERS and identified *Legionella* species as an important infectious cause [12]. However, extrasplenial abnormalities have rarely been described in *Legionella*-associated cases. Trenz and Snyder reported *Legionella*-associated CLOCC accompanied by bilateral cortical diffusion restriction and subcortical white matter FLAIR abnormalities [8]. In contrast, our patient had separate unilateral cortical and white matter lesions showing restricted diffusion, both of which ultimately resolved completely. In our literature search, we identified no published *Legionella*-associated CLOCC/MERS case with the same combination of unilateral cortical and unilateral white matter diffusion-restricted lesions.

Both systemic infection and hyponatremia may have contributed to the MRI abnormalities in our patient. Hyponatremia has been associated with reversible splenial lesions [4], and cytotoxic or intramyelinic edema has been proposed as an underlying mechanism [1,4]. The callosal, cortical, and white matter abnormalities were detected during acute *Legionella* pneumonia with marked hyponatremia and resolved as the clinical condition improved. These observations suggest that the acute systemic illness and metabolic disturbance may have provided a common background for the multifocal abnormalities; however, they do not establish whether infection, hyponatremia, or another mechanism was primarily responsible. The tissue-level mechanisms of the callosal and extrasplenial lesions may also have differed.

Other causes of the extrasplenial lesions remain possible. Peri-ictal abnormalities can produce cortical diffusion restriction [13], although no clinical seizure was witnessed; electroencephalography was not performed. Inflammatory encephalitis could not be completely excluded because the CSF showed mild pleocytosis and viral PCR testing was not performed, although the CSF findings were nonspecific. Ischemia was also considered because the extrasplenial lesions showed restricted diffusion; however, their complete resolution and the absence of a definite residual FLAIR abnormality on day 33 after admission were atypical for established cerebral infarction. Trauma and posterior reversible encephalopathy syndrome were considered less likely based on the lesion distribution and clinical setting. Osmotic demyelination syndrome was considered unlikely because the MRI abnormalities were already present on hospital day 3 while hyponatremia persisted, available sodium measurements did not suggest rapid correction, and the lesion distribution was not characteristic. The splenial CLOCC and the two extrasplenial lesions showed restricted diffusion with corresponding reductions in ADC values and ultimately resolved. Because they were detected during the same acute illness, a shared systemic trigger is possible. However, the differing temporal evolution of the lesions and the incomplete exclusion of peri-ictal, inflammatory, and ischemic mechanisms preclude establishing a common pathophysiology.

The present case has several limitations. Electroencephalography and viral PCR testing were not performed, limiting our ability to exclude peri-ictal abnormalities or viral encephalitis. Only a single CSF examination was performed, and the mild pleocytosis was nonspecific. In addition, although reduced ADC values were demonstrated quantitatively in all three lesions, their pathological basis cannot be determined from MRI findings alone. Accordingly, the precise relationship between the callosal and extrasplenial abnormalities remains uncertain.

## Conclusions

This was a case of *Legionella* pneumonia with marked hyponatremia in which a splenial CLOCC was accompanied by unilateral cortical and white matter lesions with restricted diffusion. The splenial CLOCC and white matter lesion resolved by hospital day 13, whereas the cortical lesion persisted on day 13 but resolved by day 33. The extrasplenial abnormalities were atypical of the conventional MERS type 2 pattern, and their relationship to the CLOCC and their underlying mechanisms remain uncertain. This case illustrates that reversible cortical and white matter diffusion abnormalities may occur in the setting of *Legionella* pneumonia and marked hyponatremia, but a common pathophysiology cannot be established.
